# Image Quality as an Important Confounder in Quantitative OCT Angiography: A Review with Quantitative Synthesis

**DOI:** 10.3390/medsci14040498

**Published:** 2026-08-20

**Authors:** Lilla István, Cecilia Czakó, Róbert Debreczeni, Péter Sótonyi, András Horváth, Nóra Szentmáry, Zoltán Zsolt Nagy, Illés Kovács

**Affiliations:** 1 Department of Ophthalmology, Semmelweis University, H-1085 Budapest, Hungary; istvan.lilla@semmelweis.hu (L.I.); czako.cecilia@semmelweis.hu (C.C.); szentmary.nora@semmelweis.hu (N.S.); nagy.zoltan.zsolt@semmelweis.hu (Z.Z.N.); 2 Department of Neurology, Semmelweis University, H-1085 Budapest, Hungary; debreczeni.robert@semmelweis.hu; 3 Department of Cardiovascular Surgery, Semmelweis University, H-1085 Budapest, Hungary; sotonyi.peter1@semmelweis.hu; 4 Neurocognitive Research Center, Nyírő Gyula National Institute of Psychiatry, and Addictology, H-1145 Budapest, Hungary; horvath.andras1@semmelweis.hu; 5 Department of Anatomy, Histology and Embryology, Semmelweis University, H-1094 Budapest, Hungary; 6 Faculty of Medicine, Universidade de Lisboa, 1649-028 Lisbon, Portugal; 7 Institute of Cognitive Neuroscience and Psychology, Research Centre for Natural Sciences, Hungarian Research Network, H-1117 Budapest, Hungary; 8 Experimental Ophthalmology, Saarland University, 66424 Homburg, Germany; 9 Department of Clinical Ophthalmology, Faculty of Health Sciences, Semmelweis University, H-1085 Budapest, Hungary; 10 Department of Ophthalmology, Weill Cornell Medicine, New York, NY 10021, USA

**Keywords:** OCT angiography, image quality, signal strength, vessel density, scan quality, retinal microvasculature, quantitative synthesis, standardisation

## Abstract

Background: Optical coherence tomography angiography (OCTA) provides quantitative metrics of the retinal microvasculature, most prominently vessel density (VD), that are increasingly used as biomarkers in ocular, systemic, and cerebrovascular disease. Because OCTA relies on the detection of flow-related motion contrast, image quality has emerged as a pervasive determinant of these metrics, yet its effect has been reported in fragmentary and non-comparable ways across the literature. Methods: We identified OCTA studies indexed in PubMed that examined the relationship between image quality and quantitative OCTA parameters, and we summarised their methods and findings across the macular, foveal avascular zone, and peripapillary regions and across vascular layers. We integrated directly comparable per-unit scan-quality effects using random-effects meta-analysis and separately synthesized direct cross-sectional Pearson correlations between manufacturer-reported image quality and macular vessel-density outcomes from independent healthy cohorts. Results: Higher image quality was associated with higher measured vessel density in every contributing dataset. On the Optovue scan-quality (SQ, 0–10) scale, superficial macular VD increased by 3.46% (95% CI 1.85–5.07) per SQ unit in controlled signal-attenuation experiments and by covariate-adjusted observational estimates ranging from 0.90% to 2.16% per SQ unit; a combined order-of-magnitude estimate across both estimator types was 1.64% (95% CI 1.15–2.12). Peripapillary estimates were heterogeneous and derived from only two independent cohorts; they were therefore summarised descriptively rather than pooled. Across three independent externally authored healthy cohorts reporting direct cross-sectional Pearson correlations, higher image quality was strongly associated with higher macular vessel-density outcomes (random-effects pooled r = 0.64, 95% CI 0.50–0.75; *I*^2^ = 49%). Conclusions: Image quality is an important, directional confounder of VD-based OCTA metrics whose magnitude can rival the biological or physiological signal of interest. Platform-appropriate standardisation, transparent reporting, and consideration of image quality in acquisition and analysis are therefore important for the valid interpretation of vessel-density measurements, particularly in functional and longitudinal studies.

## 1. Introduction

Optical coherence tomography angiography (OCTA) is a non-invasive, dye-free imaging technique that resolves the retinal microvasculature in three dimensions and layer by layer. It generates image contrast by detecting the motion of flowing erythrocytes across rapidly repeated cross-sectional scans, so that perfused vessels stand out against static tissue without any injected contrast agent [1]. Because it is derived from structural optical coherence tomography, which has become the most frequently performed imaging procedure in ophthalmology, OCTA is fast to acquire and, distinctively, can depict the superficial and deep capillary plexuses and the choriocapillaris separately [1]. These advantages have driven its rapid adoption in primary ocular disease such as diabetic retinopathy, retinal vascular occlusion, neovascular age-related macular degeneration, and glaucoma [1,2], and increasingly also as a window onto systemic and cerebrovascular status, including carotid artery stenosis, impaired cerebrovascular reactivity, monoclonal gammopathy, and neurodegenerative and neuroinflammatory disorders [3,4,5,6,7].

The clinical and scientific value of OCTA rests largely on its quantitative outputs. Metrics such as vessel density (VD), perfusion density (PD), vessel length density (VLD), and foveal avascular zone (FAZ) area and circularity are intended to serve as objective, reproducible biomarkers of retinal perfusion that can be tracked within an individual over time and compared across groups [8,9]. For such comparisons to be valid, the measured value must reflect the true state of the microvasculature rather than the technical conditions under which the scan was acquired. This is a demanding requirement, because the available devices, imaging protocols, segmentation algorithms, and analysis metrics differ widely and have been applied inconsistently between studies. The resulting literature is fragmented, cross-comparison and pooling of data remain difficult, and agreed minimum standards for acquisition, metrics, and reporting are still lacking, which is why methodological reviews have repeatedly called for the standardisation of OCTA imaging and reporting as a prerequisite for the field to mature [1].

Among the technical factors that threaten the validity of OCTA quantification, image quality has emerged as the most pervasive. Because motion contrast depends on an adequate signal, any reduction in signal strength, whether from media opacity, defocus, tear-film instability, poor fixation, or eye movement, lowers the apparent density of detected vessels. Studies in healthy eyes have shown consistently that VD, PD, and FAZ metrics increase as the manufacturer-reported signal-strength index rises, with strong correlations between signal strength and macular VD and comparable effects in the peripapillary region, where the influence of signal strength on VD rivals that of established structural determinants [8,10]. Controlled experiments that deliberately attenuate the beam using neutral-density or absorptive filters, defocus, or applied ointment have confirmed that this relationship is causal rather than merely associative, and have allowed per-unit correction factors for the resulting bias to be calculated [11,12,13]. Even within the range generally regarded as acceptable, small decrements in signal strength measurably lower flow parameters [12].

Adequate signal strength alone does not guarantee a usable scan. Some scans contain visible artifacts, such as vessel doubling, white-line motion artifacts, banding, blink, and segmentation or projection errors, and when these are recognised the acquisition is normally repeated [2]. Scans that appear acceptable on inspection are then characterised by a single manufacturer-specific quality value, and device makers typically regard an Optovue scan-quality score of at least 6 as the minimum acceptable quality [13]. Even this threshold is imperfect, however, because a substantial proportion of scans that pass conventional signal criteria nonetheless prove to be of non-acceptable quality on closer inspection, and projection, segmentation, and motion artifacts remain common [2,14]. Image quality is therefore not merely a source of random noise but a directional confounder that can bias both cross-sectional and longitudinal inference. In pseudoexfoliation syndrome, an apparent reduction in peripapillary VD disappeared after adjustment for signal strength, whereas a genuine parafoveal deficit persisted, so that neglecting quality could have produced both a false-positive and a missed true finding [15]. In diabetic eyes, reproducibility itself depends on image quality, and correction factors have been proposed to make follow-up measurements comparable [9,16]. The problem is most acute in functional and longitudinal paradigms, where in one neurovascular-coupling study the physiological signal of interest was of the same order of magnitude as the change in VD attributable to scan quality, so that quality and acquisition order had to be modelled explicitly to isolate the true effect [17]. These observations have motivated systematic efforts to control OCTA quality, including deep-learning models that classify image quality more accurately than the machine-reported signal strength [18,19] and international consensus frameworks such as the OSCAR-MP criteria, which codify the principal quality-degrading factors into a validated, reproducible scoring scheme [3].

Despite this growing recognition, estimates of the magnitude of the image-quality effect on VD remain scattered across heterogeneous studies and have not been quantified in a unified manner. Different studies used different quality scales, namely signal strength on a 1–10 scale, the signal-strength index on an approximately 0–100 scale, and the scan-quality value, and they applied different devices, regions, layers, and populations, and reported heterogeneous effect measures ranging from correlation coefficients to group differences by signal-strength bin to per-unit regression slopes [8,9,10,11,12,13]. No previous synthesis has integrated these disparate estimates into a common quantitative summary of how strongly and how consistently image quality biases VD-based metrics. A supplementary, targeted search for meta-analyses or systematic reviews addressing image quality as a quantitative determinant of OCTA vessel density (PubMed, terms combining ”OCT angiography” with “meta-analysis” or “systematic review” and quality-related terms) did not identify any prior quantitative synthesis of this relationship. Drawing on OCTA studies spanning healthy, diabetic, carotid-stenosis, and other cohorts, we first summarise the methods and findings linking image quality to quantitative parameters across the macular, FAZ, and peripapillary regions and vascular layers. We then express the image-quality--VD relationship on two metrics: a per-unit change in VD per unit of scan quality and a scale-independent synthesis of direct cross-sectional Pearson correlations restricted to independent healthy cohorts reporting a manufacturer-reported image-quality index and a macular vessel-density outcome. Our aim is to establish whether image quality should be regarded as an important confounder whose standardised assessment and, where validated, statistical adjustment should be considered in the interpretation of VD-based OCTA results.

## 2. Materials and Methods

### 2.1. Literature Search and Study Selection

**Figure 1:**
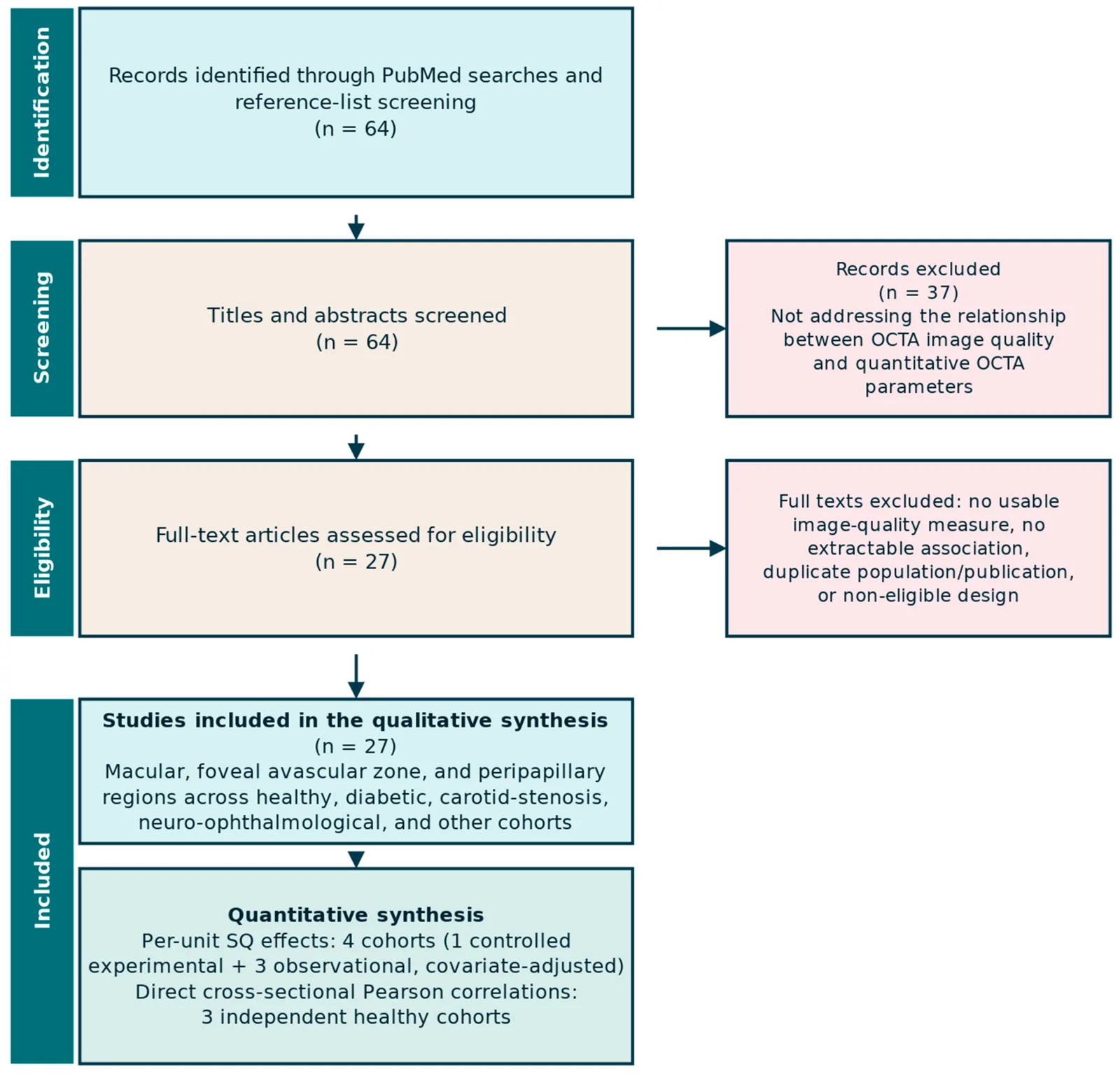
Study-selection flow diagram. Records retrieved through PubMed searches and reference-list screening were screened. The final number of studies included in the qualitative synthesis and the numbers entering each quantitative analysis are shown in the diagram. PRISMA terminology is used for reporting clarity only and does not imply full PRISMA compliance.

We searched PubMed for studies examining the relationship between OCTA image quality and quantitative OCTA parameters. Searches were conducted on 23 June and 13 August 2026. The updated search on 13 August 2026 incorporated the additional terms ‘signal strength’ and ‘signal-strength index’ and identified two further eligible studies, which were included in the qualitative synthesis and, where eligible, in the correlation synthesis. The search strategy combined ‘OCT angiography’ with terms representing image quality and its manufacturer-reported indices, including ‘retina image quality’, ‘scan quality’, ‘signal strength’, and ‘signal-strength index’. Reference lists of relevant articles were also screened to identify additional eligible studies. Title/abstract screening and full-text eligibility assessment were performed by a single reviewer, C with the final list of included studies verified by a senior co-author; a second independent screener and a formal disagreement-adjudication procedure were not used. Titles and abstracts were screened for relevance, and full texts were assessed for studies that reported quantitative OCTA metrics together with a measure of image quality (signal strength, signal-strength index, or scan quality). Studies were eligible if they reported the association between image quality and a quantitative OCTA parameter in a form usable for qualitative synthesis, and, for the quantitative pooling, if they additionally reported an effect size that could be expressed on a common scale (a per-unit slope, correction factor, or correlation coefficient with the information needed to derive its variance). The selection process is summarised in the study-selection flow diagram (Figure 1). A subset of the included cohorts originated from the present authors’ group, these studies were assessed using the same criteria applied to externally reported data. Supplementary Table S1 summarises the studies included in the qualitative synthesis, together with their OCTA platform, image-quality metric, design, and study population.

### 2.2. Study Populations and Design

The included studies used cross-sectional, prospective observational, and retrospective case-series designs, as well as controlled experimental protocols in which signal strength was deliberately manipulated in the same eyes. The populations comprised healthy volunteers [8,10,12,13,20,21], patients with non-proliferative and proliferative diabetic retinopathy [9,16,22], patients with retinal vascular occlusion, neovascular age-related macular degeneration, and other retinal disease [2,23,24], individuals with pseudoexfoliation syndrome [15], patients with neurodegenerative and neuroimmunological disorders [3,19], patients with monoclonal gammopathy [6], and patients with carotid artery stenosis [4,5,7]. Sample sizes ranged from small mechanistic studies of 10–30 eyes [12,13,20,24] to larger healthy and diabetic cohorts of 84–446 eyes [8,9,10,16,21], dedicated carotid-stenosis cohorts of up to 112 eyes in 56 patients [4,5,7], and multicentre quality analyses processing several thousand scans [14,22]. The anatomical regions examined were the macula, the FAZ, and the peripapillary region, and where reported the superficial capillary plexus, deep capillary plexus, and choriocapillaris were analysed separately [8,9,10,12,13,15,20].

### 2.3. Devices, Quality Indices, and Quantitative Parameters

The studies employed several commercial OCTA platforms, which is itself a source of heterogeneity in the reported metrics and quality indices. In this review, ‘image quality’ is used as an umbrella term, whereas SQ, SSI, and SS refer to distinct manufacturer-specific metrics and are not treated as numerically interchangeable. The most frequently used systems were the spectral-domain Optovue RTVue XR Avanti with AngioVue software [4,5,6,7,9,13,16,17], the Zeiss Cirrus AngioPlex [2,8,10,18], and swept-source devices, and several studies directly compared spectral-domain and swept-source instruments [12]. Image quality was expressed through the manufacturer-specific, built-in indices: the scan-quality (SQ) value and, on some software versions, the signal-strength index (SSI) in the Optovue system [9,13], and the signal strength (SS) on a 1–10 scale in the Cirrus system [8,10].

The quantitative outcome parameters were defined as follows. Vessel density (VD) is the proportion of the measured area occupied by perfused vessels, expressed as a percentage and computed from the binarised en face flow image. Perfusion density (PD), reported by some software packages, is a closely related area-based measure of the fraction of tissue perfused. Vessel length density (VLD), also termed vessel skeleton density, is the total length of the vessel skeleton per unit area and is less sensitive to vessel calibre than VD. FAZ area and circularity quantify the size and shape of the central avascular region, and choriocapillaris flow deficits (CCFD) quantify the area of absent choriocapillaris signal. These parameters were derived from the binarised and skeletonised en face angiograms of the relevant slab, and quantification was performed either with the manufacturers’ built-in software, for example Optovue AngioAnalytics or the Cirrus AngioPlex software, or with custom analysis tools, as specified in each source study [8,9,12,13,16,20,24]. Although FAZ metrics and choriocapillaris flow deficits were recorded where reported, too few studies provided a poolable image-quality effect on these parameters, so the quantitative synthesis was confined to vessel density; FAZ and choriocapillaris findings are summarised qualitatively only. Because the exact algorithm, software version, and slab definition differ between platforms, values are not numerically interchangeable across devices, a point we accounted for in the synthesis.

### 2.4. Reported Approaches to Manipulating and Controlling Image Quality

To probe the causal relationship between image quality and OCTA metrics, several studies induced deterministic signal loss in the same eyes by inserting neutral-density or absorptive filters of varying optical density into the optical path, or through deliberate defocus and topical ointment application [11,12,13,20]. These paradigms allowed the VD-versus-signal-strength slope and per-unit correction factors to be estimated. Reliability was characterised by repeated scanning within a session, between visits, and over the longer term [9,16,21]. To standardise quality control, several groups trained supervised deep-learning classifiers on expert-graded images [14,18,19], and an international consensus framework, the OSCAR-MP criteria, was developed and validated across multiple centres [3]. These device-, algorithm-, and consensus-based approaches represent the methodological toolkit that the primary studies used to characterise and mitigate the image-quality effect, and they are summarised here as the background against which our quantitative synthesis is set.

### 2.5. Statistical Analysis of the Present Synthesis

Two complementary syntheses were performed. Per-unit scan-quality effects on vessel density (VD) were pooled separately by estimator type: correction factors from controlled signal-attenuation experiments [13] and covariate-adjusted regression coefficients from observational studies [4,5,6,17]. As Optovue SQ and SSI are non-interchangeable scales, only SQ-based estimates were pooled quantitatively; SSI-based results were assessed qualitatively. Superficial macular VD was the primary endpoint. Peripapillary all-vessel and small-vessel outcomes were summarised separately as descriptive, non-pooled findings because the available estimates were sparse and correlated within cohort. Correlation-only and repeatability studies were used for corroboration [11,12,20,21]. Estimates were combined by inverse-variance random-effects models, with standard errors taken from the original reports or derived from 95% confidence intervals. For the correlation synthesis, we included only direct cross-sectional Pearson correlations between a manufacturer-reported image-quality index and a macular vessel-density outcome from independent healthy cohorts. One effect estimate was selected per independent cohort. Correlations were transformed to Fisher z values, pooled using DerSimonian--Laird random-effects models with variance 1/(*n* − 3), and back-transformed to the correlation scale. Partial correlations, rank correlations, controlled experimental within-eye associations, and correlations between within-eye changes in image quality and vascular measurements were not combined with this cross-sectional Pearson-correlation estimand. Derived slopes without reconstructable confidence intervals were used for directional corroboration only and were not pooled. Because only three independent cohorts met these criteria, the pooled correlation was regarded as a supportive, scale-independent summary of directional association rather than as a universal device-independent correction factor. Random-effects models were pre-specified because of differences in device, region, vascular layer, and population. Controlled correction factors and covariate-adjusted observational regression coefficients were analysed as distinct estimator strata because they quantify different aspects of the quality–VD relationship: experimentally induced maximal signal-related distortion and residual association after adjustment, respectively. Where a combined analysis was performed, it was treated as a secondary descriptive order-of-magnitude estimate rather than as a single exchangeable biological effect. Heterogeneity was described using Cochran’s Q and *I*^2^, with *I*^2^ > 60% indicating substantial heterogeneity; *k* denotes the number of estimates. No formal risk-of-bias instrument was applied to the included studies. For the observational, covariate-adjusted cohorts, the ROBINS-I (Risk Of Bias In Non-randomised Studies of Interventions) tool would in principle be the appropriate instrument; however, no single risk-of-bias instrument was applied across all included studies because the evidence base comprised heterogeneous controlled signal-attenuation experiments, observational cohorts, repeatability studies, and retrospective clinical series. Although ROBINS-I may be informative for some observational analyses, it was not considered directly applicable to all designs represented in the present synthesis. To facilitate structured appraisal, the evidence tables report study design, population, device, quality metric, anatomical outcome, and estimator type for each quantitative estimate.

## 3. Results

### 3.1. Signal Strength and Macular Vessel Density in Healthy Eyes

**Figure 2:**
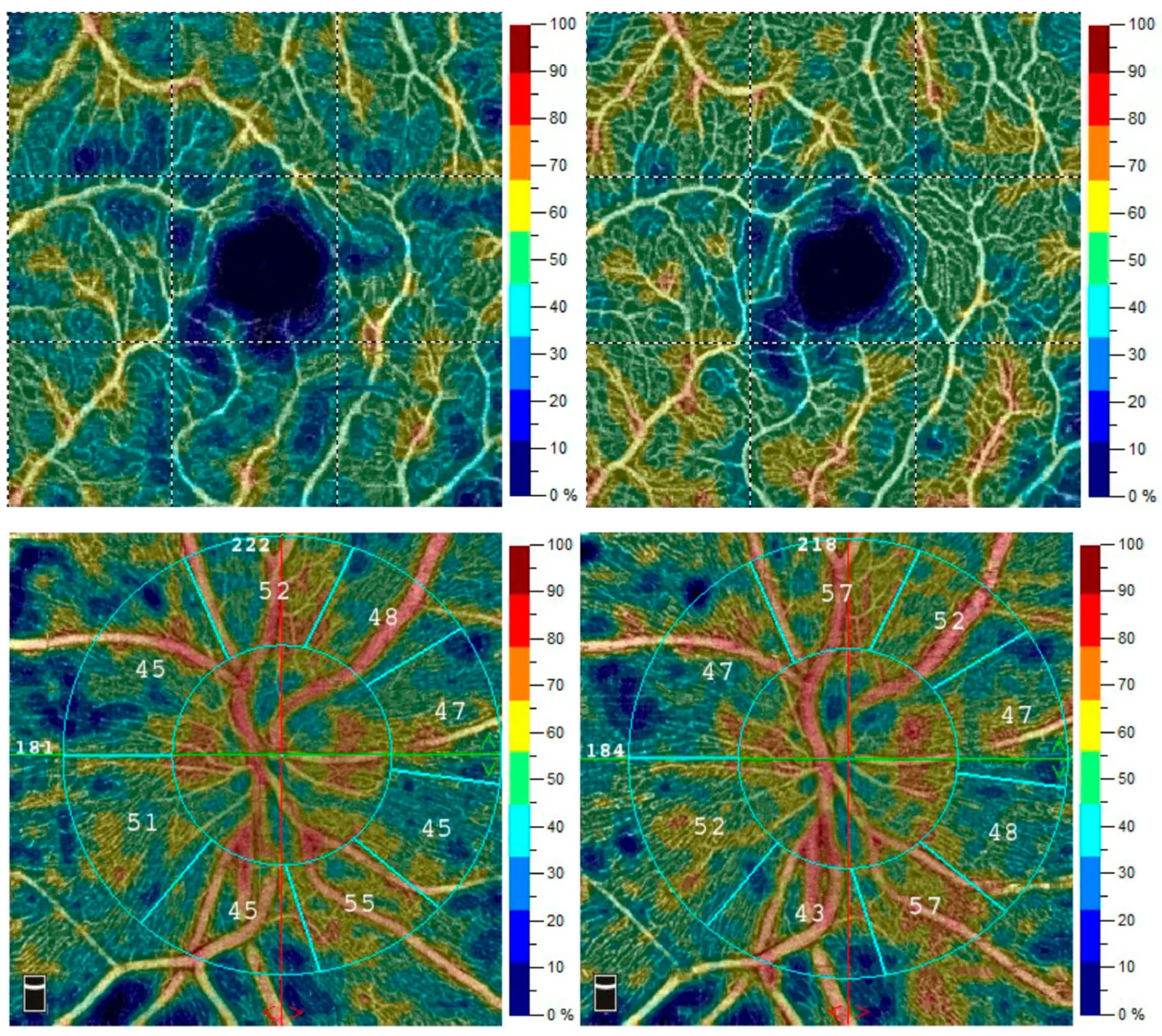
Same-eye superficial macular maps (top: SQ 6, left; SQ 8, right) and peripapillary maps (bottom: SQ 6, left; SQ 9, right) on the standard 0–100% en-face scale. Higher SQ shows denser, more continuous capillaries and higher sector densities, illustrating quality-related pseudo-flow change.

In healthy eyes, whole-image VD, PD and FAZ area increased significantly with signal strength across the range of 7 to 10 (*p* < 0.001), although no significant difference was observed between signal strengths of 9 and 10. These parameters correlated with signal strength as follows: VD, r = 0.668; PD, r = 0.671; and FAZ area, r = 0.570 (all *p* < 0.001), collectively supporting a minimum acceptable signal strength of 9 [8]. Comparable findings were reported across two commercial platforms, where VD declined linearly as signal strength decreased, with the steepest decline occurring after filter-induced attenuation; both signal strength and age were identified as determinants of inter-individual VD variance [11]. In an independent cohort of 30 eyes from 15 healthy participants, absorptive filters yielded macular and peripapillary correction factors of 2.27–3.92% per SQ unit [13]. Consistent with these observations, the application of neutral-density filters resulted in decreased superficial VD and VLD as signal strength fell from 10 to 8.2 [12]. Controlled artificial-degradation experiments similarly demonstrated a marked reduction in superficial macular VD under conditions of poorer image quality [20]. Figure 2 illustrates this quality-dependent gradient.

### 3.2. Signal Strength and Vessel Density in the Peripapillary Region

In a cohort of 259 healthy eyes, mean VD and PD increased with signal strength across the 7–10 range (*p* < 0.001). Mean VD was additionally correlated with age (partial r = 0.133), retinal nerve fibre layer (RNFL) thickness (partial r = 0.169), cup–disc ratio (partial r = −0.481), and signal strength (partial r = 0.413) [10]. Short-term and long-term (≥6-month) repeatability were characterised by coefficients of variation of 2.94–4.22% (ICC 0.840–0.934) and 2.73–3.84% (ICC 0.737–0.934), respectively, with both axial length and mean signal strength contributing to long-term repeatability (B = 2.028; *p* < 0.001) [21].

### 3.3. Reproducibility and Image Quality in the Diabetic Population

In 54 eyes from 27 patients, intraclass correlation coefficients exceeded 0.90 for both within-visit and between-visit measurements. The coefficient of variation for FAZ area exceeded that of VD, and between-visit VD repeatability was 4.53% (95% CI 3.72–5.79); notably, variability in signal strength correlated with variability in VD [16]. In a larger cohort of 100 eyes from 50 patients with non-proliferative diabetic retinopathy, the signal strength index (SSI) ranged from 30 to 85 and correlated positively with retinal capillary VD while also improving measurement repeatability. The resulting per-SSI-unit correction factors were 0.22% (95% CI 0.20–0.24) for 3 × 3 mm macular VD and 0.23% (95% CI 0.21–0.26) for perifoveal VD [9].

### 3.4. Image Quality and Interpretability in Retinal Diseases

In a sample of 75 eyes comprising patients with diabetic retinopathy, retinal vascular occlusion, neovascular age-related macular degeneration (AMD), and healthy controls, projection artifacts were present in every eye beneath the superficial layer, whereas segmentation and motion artifacts were observed in 55% and 49% of eyes, respectively. Both segmentation artifacts and macular oedema were significantly associated with reduced interpretability (both *p* < 0.01) [2]. In retinal vein occlusion, averaging five en face acquisitions reduced VLD in both the superficial layer (15.5 ± 2.5 vs. 17.8 ± 2.4/mm; *p* = 0.05) and the deep layer (16.2 ± 1.4 vs. 18.5 ± 1.6/mm; *p* = 0.003), indicating that image averaging itself can alter quantitative outcomes [24]. In neovascular AMD, manual segmentation correction improved image quality in 55% of acquisitions and increased the proportion of good-quality images from 63% to 83% [23].

### 3.5. Correction for Signal Strength as a Prerequisite for Interpretation

In pseudoexfoliation syndrome, raw peripapillary VD was lower in cases than in controls (58.2% vs. 58.8%; *p* = 0.04); however, this difference was no longer significant following adjustment for intraocular pressure and signal strength (*p* = 0.39). By contrast, adjusted parafoveal VD remained significantly lower in cases relative to controls (44.3% vs. 46.8%; *p* = 0.008), highlighting the parameter-specific nature of confounding by image quality [15].

### 3.6. Retinal OCTA as a Marker of Systemic and Cerebrovascular Diseases

In 112 eyes from 56 patients with significant carotid stenosis, lower estimated glomerular filtration rate, hypertension, and carotid occlusion were each associated with reduced retinal blood flow, whereas statin use and carotid surgery were associated with improved ocular microcirculation [4]. VD decreased with advancing age and was higher in eyes with a non-compromised Circle of Willis configuration, increasing further following carotid endarterectomy; scan quality remained a significant covariate throughout the mixed-model analysis [5]. Impaired cerebrovascular reactivity was likewise associated with reduced peripapillary VD [7]. In monoclonal gammopathy, superficial VD remained significantly lower than in controls even after adjustment for scan quality (44.54% vs. 46.62%; *p* < 0.05) [6].

### 3.7. Prevalence of Artifacts and Automated Quality Assessment

Among 4555 images acquired from 546 patients with signal strength ≥ 6, approximately 21% were classified as non-acceptable for analysis [14]. In multicentre diabetic trials, 61% of scans were graded as good quality; low SSI on the AngioVue platform, excessive motion artifact on AngioPlex, and younger patient age were each associated with reduced image quality [22]. For automated quality assessment, a ResNet152 convolutional neural network achieved area-under-the-curve (AUC) values of 0.99 and 0.97 for identifying low- and high-quality images, respectively, substantially outperforming machine-reported signal strength (AUC 0.82 and 0.78) [18]; Convolutional neural network-based grading was similarly reliable in neurodegenerative disease cohorts [19]. The OSCAR-MP framework defines seven distinct quality-degrading factors [3], and recent methodological reviews advocate for standardised acquisition protocols, quality metrics, and reporting practices across the field [1,25].

### 3.8. Image Quality in Functional and Longitudinal OCTA

In 22 healthy older adults, light adaptation was associated with an increase in peripapillary small-vessel VD of +1.30 (*p* = 0.046), whereas macular VD was independently affected by scan quality (β = 1.62; *p* < 0.001). These findings indicate that explicit modelling of scan quality and acquisition order is necessary to isolate the underlying physiological signal from quality-related confounding [17].

### 3.9. Pooled Per-Unit Effect of Scan Quality on Vessel Density

**Figure 3:**
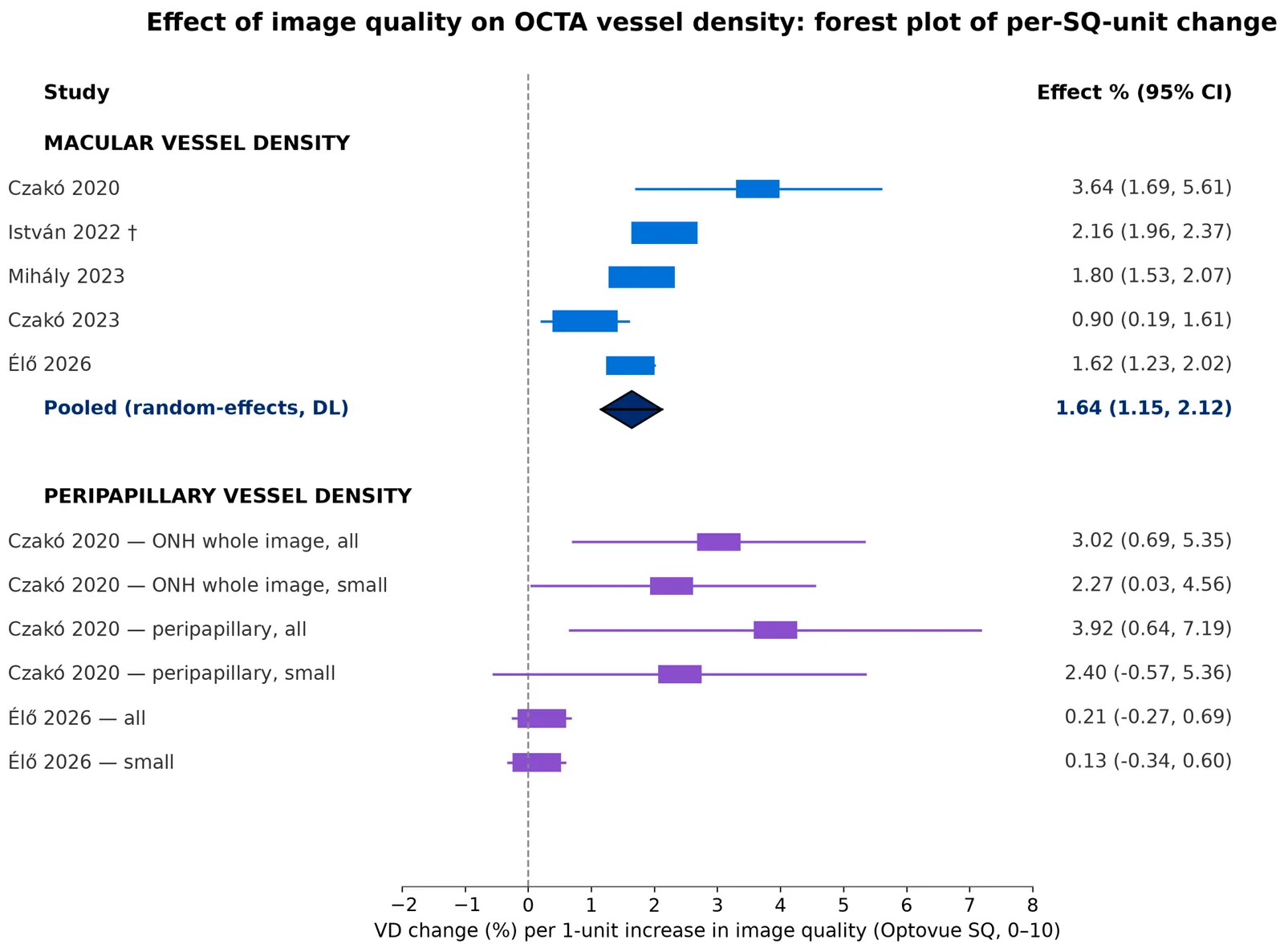
Per-unit SQ (0–10) effects on OCTA vessel density. Points show percentage change per SQ unit with 95% confidence intervals. The diamond shows the random-effects superficial macular pool. Peripapillary all-vessel and small-vessel estimates are displayed individually and were not pooled because Czakó 2020 reported correlated whole-image and peripapillary estimates from the same cohort, while only two independent cohorts were available overall [4,5,6,13,17].

For superficial macular density, all per-unit estimates were positive in direction (Table 1; Figure 3). The coefficients derived from the carotid-stenosis, monoclonal-gammopathy, and functional cohorts [4,5,6,17] were expressed on the same SQ scale and outcome as the controlled correction factor reported by Czakó [13]. Controlled experiments yielded an estimate of 3.46% (95% CI 1.85–5.07) per SQ unit, whereas covariate-adjusted observational estimates ranged from 0.90% to 2.16% per SQ unit. The combined exploratory estimate across these sources was 1.64% (95% CI 1.15–2.12; Cochran Q = 9.32, df = 3; *I*^2^ = 68%), with a Hartung-Knapp-Sidik-Jonkman (HKSJ) interval of 0.556–2.714%. Substituting the alternative carotid-stenosis estimate yielded a revised figure of 1.80%, whereas controlled data alone reproduced the original estimate of 3.46% (95% CI 1.85–5.07) [4,5,6,13,17].

For peripapillary VD, Czakó reported controlled correction factors of 3.02% and 2.27% per SQ unit for whole-image all-vessel and small-vessel VD, respectively, and 3.92% and 2.40% for the corresponding annular outcomes, although the small-vessel annular estimate did not reach statistical significance [13]. By contrast, Élő reported covariate-adjusted coefficients of 0.21% for all-vessel and 0.13% for small-vessel peripapillary VD [17]. Because the whole-image and annular estimates from Czakó were derived from the same experimental cohort and were therefore not statistically independent, and because only two independent cohorts were available overall, a pooled peripapillary effect estimate was not calculated [13,17]. These findings should accordingly be regarded as descriptive rather than as evidence for a single generalisable peripapillary correction factor.

### 3.10. Direct Cross-Sectional Correlations Between Image Quality and Macular Vessel Density

**Figure 4:**
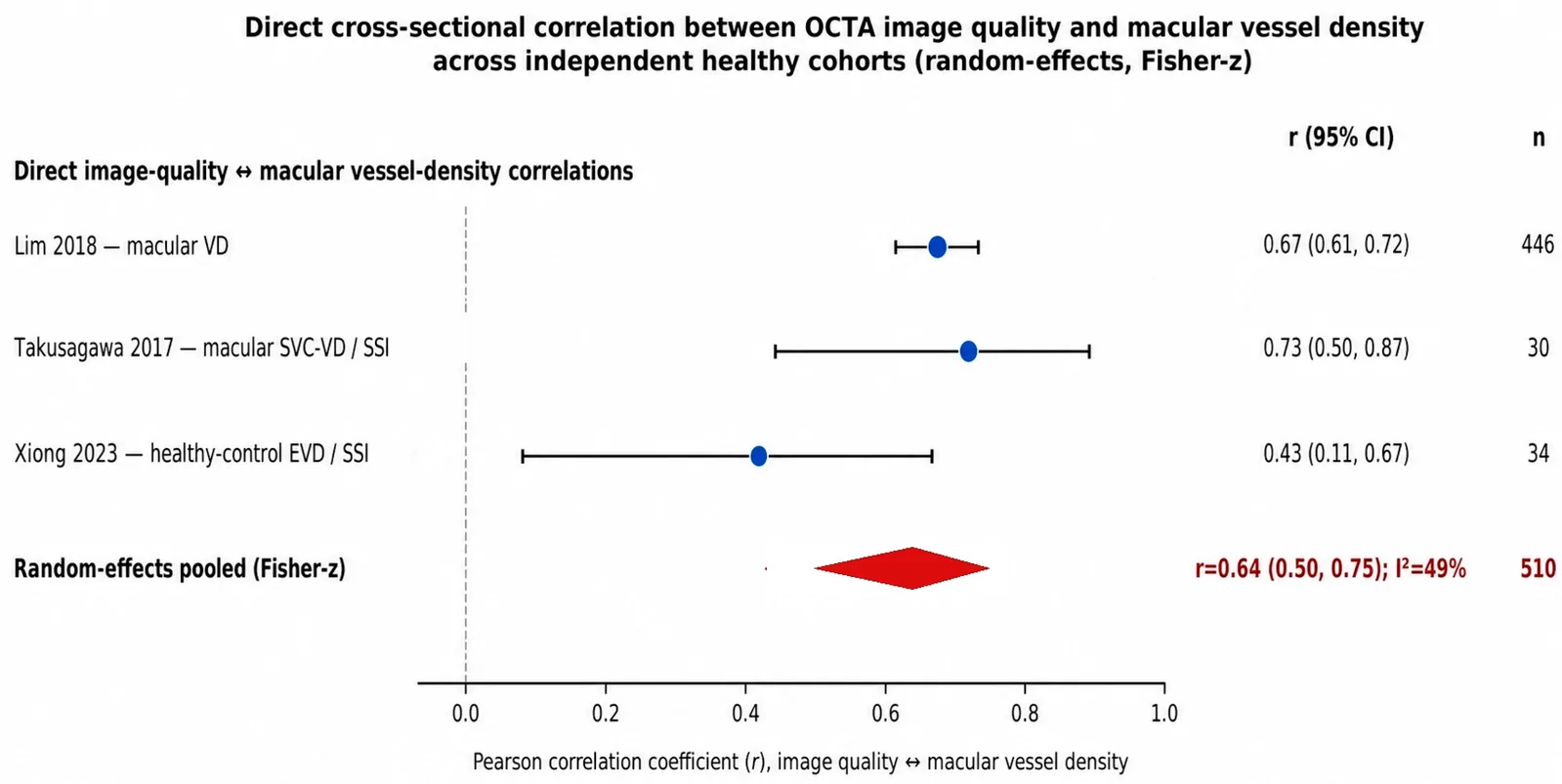
Direct cross-sectional correlations between manufacturer-reported image quality and macular vessel-density outcomes in independent healthy cohorts. Points represent study-specific Pearson correlations and 95% confidence intervals after Fisher z-transformation; the diamond represents the DerSimonian--Laird random-effects pooled correlation (r = 0.64, 95% CI 0.50–0.75; k = 3; *I*^2^ = 49%). Studies reporting partial correlations, within-eye change correlations, repeated-measurement effects, controlled experimental associations, or non-equivalent vascular outcomes were not included in the pooled estimate [8,26,27].

Three independent externally authored healthy cohorts reported direct cross-sectional Pearson correlations between manufacturer-reported image quality and macular vessel-density outcomes. Lim et al. reported a correlation of r = 0.668 between Cirrus signal strength and macular vessel density in 446 healthy eyes [8]. Takusagawa et al. reported a correlation of r = 0.731 between signal-strength index and fixed-threshold superficial vascular-complex vessel density in 30 healthy eyes [26], whereas Xiong et al. reported r = 0.434 between signal-strength index and commercial extrafoveal vessel density in 34 healthy control eyes [27]. Random-effects synthesis after Fisher z-transformation yielded a pooled correlation of r = 0.64 (95% CI 0.50–0.75; k = 3; Cochran Q = 3.94; *I*^2^ = 49%; τ^2^ = 0.018, Table 2, Figure 4). This summary was restricted to direct cross-sectional Pearson correlations in independent healthy cohorts. Partial correlations, within-eye change correlations, repeated-measurement associations, experimental attenuation data, and correlations involving non-equivalent flow outcomes were not combined with this estimand. The pooled value should therefore be interpreted as scale-independent corroboration of a positive macular image quality—vessel density association, not as a device independent correction factor.

## 4. Discussion

This work assembled the heterogeneous evidence linking OCTA image quality to quantitative vessel-density metrics and integrated it into two complementary quantitative syntheses: a per-unit-of-quality slope pool and a scale-independent pooled correlation [28,29]. The important finding is unambiguous and directionally consistent across every contributing dataset. Higher image quality is associated with higher measured vessel density, and the magnitude of this dependence is large enough to rival the biological and physiological signals that quantitative OCTA is intended to detect.

The most secure conclusion of the present synthesis is that scan quality exerts a substantial, positive, and reproducible influence on superficial macular vessel density. What gives this estimate its weight is not the point value itself but the convergence between two methodologically distinct approaches within our own research programme (a controlled signal-attenuation paradigm and observational covariate-adjusted models), rather than between fully independent research groups. On one side lie the deterministic signal-loss experiments, in which the same eyes are imaged repeatedly while transmittance is attenuated by absorptive filters, defocus, or applied ointment; these designs isolate the pure quality effect and establish that the relationship is causal rather than merely associative. On the other side lie the large observational cohorts, in which scan quality was never the exposure of interest but was carried in multivariable mixed models as a nuisance covariate, and in which its regression coefficient nonetheless emerged as sizeable and highly significant. That two such different approaches, applied to different populations and different clinical questions, converge on a common effect on a common scale is the strong internal evidence that the association is not solely a feature of a single experimental paradigm, while independent replication across devices, populations, and research groups remains necessary to establish its broader generalisability.

The residual heterogeneity in this pool is itself informative and should not be read as disagreement. The direction of effect is uniform across every contributing cohort; the dispersion arises because the experimentally isolated correction factors tend to be larger than the covariate-adjusted field coefficients. This is exactly the pattern one would predict on mechanistic grounds. A controlled attenuation experiment measures the full sensitivity of the binarised flow signal to loss of contrast, uncontaminated by the compensatory selection that operates in routine practice, whereas an observational coefficient is estimated after other correlates of density have already absorbed part of the variance and after low-quality scans have often been excluded at acquisition. The controlled estimate therefore behaves as an upper bound on the true bias, and the adjusted field estimate as a more conservative, real-world figure. Rather than undermining the pooled result, this gradient clarifies how it should be used: the larger correction factor is appropriate when reconstructing the maximal possible distortion, and the smaller regression coefficient when estimating the residual bias that survives conventional quality screening. The numerically similar order of magnitude of the SSI- and SQ-based estimates is directionally consistent with the overall finding; however, no formal numerical conversion was performed because the manufacturer does not define a validated correspondence between these device-specific indices.

Although several controlled experimental peripapillary correction factors were positive and statistically significant, the covariate-adjusted observational coefficients from the functional cohort were near null and imprecise. Because these estimates were sparse, derived from only two independent cohorts, and partly correlated within cohort, they were not pooled and do not support a single generalisable peripapillary correction factor. The reason is not that peripapillary density is immune to image quality, for the controlled experiment clearly showed that it is not; rather, the small number of poolable cohorts and the marked divergence between the substantial controlled correction factors and the near-null coefficients from the functional cohort left the summary underpowered and unstable. Several features of the optic-nerve-head region plausibly contribute to this behaviour, including the greater influence of large vessels, the steeper local anatomy, the different segmentation slabs, and the sensitivity of peripapillary metrics to axial length and disc morphology [30,31,32,33,34]. Whatever the mechanism, the practical message is unambiguous: a single pooled peripapillary correction cannot yet be recommended, and the region should be treated as a distinct measurement problem requiring dedicated, adequately powered, device-specific study rather than an extrapolation from macular findings.

The per-unit slope pool is constrained by the small number of studies reporting directly comparable coefficients. The expanded evidence base included additional externally authored correlation-based studies. To avoid combining non-equivalent estimands, the present correlation synthesis was restricted to three independent healthy cohorts reporting direct cross-sectional Pearson correlations between manufacturer-reported image-quality indices and macular vessel-density outcomes. The pooled association was strong (r = 0.64, 95% CI 0.50–0.75), although the moderate heterogeneity reflects differences in OCTA platform, quality-index scale, scan area, thresholding method, and precise macular vessel-density endpoint. This correlation should be interpreted as scale-independent corroboration that higher image quality is associated with higher measured macular vessel density across devices, rather than as a universal numerical correction factor. The supplementary search also identified additional partial correlations, repeated-measurement associations, and quality-adjusted regression coefficients, but these estimate different relationships and are therefore summarized separately rather than merged into the direct cross-sectional correlation pool.

This synthesis extends the existing literature in three concrete ways. First, it is the first quantitative synthesis, to our knowledge, to combine two complementary lines of evidence on this question: a per-unit slope pool derived from the authors’ own carotid-stenosis, gammopathy, and functional cohorts, and an independently derived pooled direct cross-sectional correlation of r = 0.64 (95% CI 0.50–0.75) drawn from three externally authored healthy cohorts [8,26,27]. Second, it formally distinguishes controlled-experiment estimates (maximal induced distortion) from covariate-adjusted observational estimates (residual real-world bias) at the level of individual anatomical parameters, including the previously conflated optic-nerve-head whole-image and peripapillary-annulus parameters, a distinction not made explicit in any single primary study. Third, it applies conservative interval estimation to the small-k per-unit synthesis and explicitly separates non-independent or non-equivalent correlation estimates from the direct cross-sectional correlation summary. This methodological distinction prevents correlated device-specific effects, partial correlations, repeated-measurement associations, and non-equivalent vascular outcomes from being presented as a single pooled correlation.

The importance of this correlation becomes clearer when it is set beside the other determinants of vessel density that clinicians already take seriously [35]. In healthy peripapillary tissue, signal strength was a stronger correlate of density than either age or nerve-fibre-layer thickness and approached the influence of the cup–disc ratio, the very parameter that dominates structural glaucoma assessment [36,37]. In other words, a purely technical acquisition variable is comparable in influence to the anatomical features that the metric is meant to interrogate. Non-pooled slopes derived from binned group means and from the single inter-individual analysis point in the same direction and the same order of magnitude, and although they were deliberately kept out of the formal pool because their group-mean origin conflates physiological with technical variance, their concordance reinforces rather than qualifies the conclusion.

These pooled estimates formalise and quantify what individual studies had previously reported only qualitatively or in isolation. It has long been recognised that reduced signal strength lowers density and perfusion metrics, degrades repeatability, and can drag the intraclass correlation of macular density from good to poor [38,39]. The contribution here is to move from a collection of single-study associations to a stable, cross-platform effect size, and to do so while reconciling two literatures that had seldom been considered together, namely the experimental signal-attenuation studies and the observational cohorts in which quality entered only as a covariate. The agreement between estimates derived on different manufacturer quality indices, despite the absence of a validated numerical conversion between these scales, further supports the interpretation that the direction of the image-quality effect is not confined to any one proprietary quality score.

The interpretive consequences echo, and now explain, a series of earlier cautionary reports. Because a difference of only one or two quality units between visits can shift macular density by an amount comparable to a genuine microvascular change, uncorrected quality differences are capable of producing spurious longitudinal progression and false cross-sectional group differences alike. This is not a theoretical worry. In pseudoexfoliation syndrome an apparent peripapillary deficit disappeared once signal strength was accounted for, while a true parafoveal deficit survived adjustment, so that neglecting quality would have produced simultaneously a false-positive and a preserved true finding. In a neurovascular-coupling paradigm the quality-driven variation in macular density was of the same order as the physiological adaptation signal under study, obliging the investigators to model quality and acquisition order explicitly before the true effect could be seen. The pooled effect size derived here supplies the quantitative rationale that makes such adjustments decisive rather than cosmetic. The same logic governs the rapidly expanding use of retinal OCTA as a biomarker of systemic and cerebrovascular disease [40,41,42,43,44], where any between-group difference in density must first be defended against the rival explanation of differential image quality before it can be attributed to carotid, haematological, or cerebrovascular pathology.

Our findings also contextualise and reinforce the methodological response now developing in the field. Comprehensive reviews have called for the standardisation of acquisition, metrics, and reporting as a precondition for cross-study comparability, and consensus frameworks now codify the principal quality-degrading factors into validated scoring schemes [45,46,47]. The recurring observation that artifacts remain common even in scans that pass conventional signal thresholds shows that manufacturer-reported signal strength is a necessary but insufficient guarantee of quality [48,49], which is precisely why automated deep-learning classifiers, standardised acquisition and averaging protocols [50,51], and manual segmentation correction [52,53,54,55] have all been advanced as complementary safeguards. What the present synthesis adds is the missing quantitative premise: by converting the qualitative claim that image quality matters into a specific bias magnitude, these safeguards are increasingly relevant to density-based analysis, particularly in functional and longitudinal designs, although their specific implementation should remain device- and context-dependent.

Several limitations should be noted. Study screening and full-text eligibility assessment were performed by a single reviewer, with the final included-study list verified by a senior co-author; a second, independent screener and formal disagreement-adjudication were not used, which is a limitation relative to PRISMA-style dual screening. The search was restricted to PubMed and reference-list screening; although the updated strategy incorporated both general image-quality terminology and manufacturer-specific indices (signal strength, signal-strength index), relevant studies indexed exclusively in other databases may still have been missed, which is likewise acknowledged as a limitation of the search strategy. Formal assessment of publication bias (funnel-plot asymmetry, Egger’s test) was not feasible given the small number of estimates in each pool, and small-study effects cannot be excluded. The per-unit slope pool rests on a small number of cohorts, is dominated by a single device platform, and shows substantial heterogeneity, so its summary should be read as an order-of-magnitude quantification rather than a definitive meta-analytic effect. The direct Pearson-correlation synthesis was restricted to three independent healthy cohorts and therefore remains imprecise despite moderate statistical heterogeneity. Its contributing studies used different device platforms, manufacturer-specific quality indices, scan sizes, and vessel-density algorithms; the pooled correlation should consequently be regarded as supportive directional evidence rather than a transportable device-independent effect size. We deliberately excluded from the pooled estimates the slopes derived from signal-strength-binned group means and the single inter-individual slope, because their group-mean origin conflates physiological and inclusion-related variation with the pure quality effect and no valid confidence interval could be reconstructed for them; these were used only for directional corroboration. Finally, this is a synthesis of published aggregate data rather than an individual-participant-data meta-analysis, and access to raw data would permit more rigorous harmonisation of scales, layers, and covariates than aggregate reporting allows. We note that the per-unit slope pool, including the controlled signal-attenuation estimate central to our conclusions, is drawn entirely from our own research group’s prior cohorts. While we are not aware of any unpublished null results from this line of work that would have been excluded, we cannot rule out the possibility of within-group publication or selection bias, and we flag independent external replication of the per-unit slope/correction-factor relationship as a priority for future research. Independent corroboration is available from the scale-independent correlation pool, which draws entirely on externally authored cohorts and shows a comparable direction and order of magnitude of association. The absence of a formal study-level risk-of-bias assessment limits the certainty with which individual quantitative estimates can be weighted and should be considered when interpreting the pooled and descriptive findings.

Several priorities follow directly from these findings. Primary studies should routinely report the per-unit quality–density relationship on a common, explicitly stated scale, with confidence intervals and ideally alongside a controlled signal-attenuation calibration, so that future syntheses are no longer confined to a handful of poolable estimates; published conversion factors between the major quality indices and across device platforms would materially improve comparability. Adequately powered, device-specific studies are especially needed for the peripapillary region, where the present pools were inconclusive. Correction strategies, whether explicit per-unit factors, covariate adjustment, or model-based residualisation, should be validated prospectively and standardised, and their use should be paired with validated quality-scoring frameworks and automated quality gates rather than reliance on manufacturer signal strength alone. Longitudinal and functional protocols should pre-specify image quality and acquisition order as covariates, given that the quality effect can equal the physiological signal of interest. Ultimately, an individual participant-data metaanalysis and prospective multicentre datasets acquired under harmonised protocols would allow the field to advance from an order-of-magnitude estimate to a precise, device- and region specific correction. Until then, the present synthesis establishes that image quality is already, on the basis of existing evidence, an important confounder in functional and longitudinal OCTA studies, and that its uniform standardisation of acquisition, transparent reporting of quality indices, and, where validated for the specific platform, quantitative correction of the image-quality effect are therefore important considerations for the valid interpretation of vessel-density measurements. Looking ahead, dedicated deep-learning models trained specifically to grade OCTA image quality offer a scalable route to flag or exclude low-quality scans before vessel-density quantification [56], further reducing image quality as a residual confounder in functional and longitudinal studies.

## 5. Conclusions

Across every dataset examined, higher image quality was associated with higher measured vessel density, and the magnitude of this dependence was large enough to rival the biological and physiological signals that quantitative OCTA is intended to detect. On the Optovue scan-quality scale, each additional unit was associated with an increase in superficial macular vessel density of roughly 1.6–3.5% across the controlled and observational estimates. In three independent healthy cohorts reporting direct cross-sectional correlations, higher image quality was strongly associated with higher macular vessel density (pooled r = 0.64, 95% CI 0.50–0.75), although this scale-independent summary should not be interpreted as a universal correction factor across devices or algorithms. The peripapillary region behaved differently: the available pools were underpowered and inconclusive, so it should be treated as a distinct measurement problem rather than an extrapolation from the macula. Taken together, these findings establish image quality as an important, directional confounder of vessel-density–based OCTA metrics rather than a source of random noise. For cross-sectional comparisons, longitudinal monitoring, functional paradigms, and the growing use of retinal OCTA as a systemic and cerebrovascular biomarker, uniform standardisation of acquisition, transparent reporting of quality indices, and, where validated for the specific platform, quantitative correction of the image-quality effect are therefore important considerations for the valid interpretation of vessel-density measurements. Between-visit changes in macular vessel density smaller than roughly 2–3% should be interpreted cautiously with respect to scan-quality variation, and quality matching or exclusion criteria should be considered and validated according to the specific device and protocol used, rather than applied as a fixed universal threshold. Realising the full biomarker potential of OCTA will ultimately require individual participant data metaanalyses and prospective, multicentre datasets acquired under harmonised protocols, enabling the field to advance from an order-of-magnitude estimate to precise, device- and region-specific correction.

## Figures and Tables

**Table 1:** Per-unit image-quality effects on OCTA vessel density. β, scan-quality coefficient from a multivariable mixed-effects/GEE model; CF, per-unit correction factor from controlled signal loss.

Study (Ref)	Population (n)	Device/Quality Index	Region and Layer	Estimate Type	Effect per 1 Quality Unit (95% CI)	Significance	Design Type
Czakó 2020 [13]	30 eyes/15 healthy	Optovue/SQ (0–10)	Macular superficial layer	CF	3.64% (1.69–5.61)	*p* < 0.001	Controlled experimental
Czakó 2020 [13]	30 eyes/15 healthy	Optovue/SQ (0–10)	Macular deep layer	CF	3.05% (0.15–5.94)	*p* = 0.03	Controlled experimental
Czakó 2020 [13]	30 eyes/15 healthy	Optovue/SQ (0–10)	Peripapillary—ONH whole image, all vessels	CF	3.02% (0.69–5.35)	*p* = 0.01	Controlled experimental
Czakó 2020 [13]	30 eyes/15 healthy	Optovue/SQ (0–10)	Peripapillary—ONH whole image, small vessels	CF	2.27% (0.03–4.56)	*p* = 0.04	Controlled experimental
Czakó 2020 [13]	30 eyes/15 healthy	Optovue/SQ (0–10)	Peripapillary—annulus, all vessels	CF	3.92% (0.64–7.19)	*p* = 0.01	Controlled experimental
Czakó 2020 [13]	30 eyes/15 healthy	Optovue/SQ (0–10)	Peripapillary—annulus, small vessels	CF	2.40% (−0.57 to 5.36)	*p* = 0.11 (NS)	Controlled experimental
István 2022 [4]	112 eyes/56 carotid stenosis	Optovue/SQ (0–10)	Macular superficial layer	β	2.16% (1.96–2.37)	*p* < 0.001	Observational, covariate-adjusted
Mihály 2023 [5]	112 eyes/56 carotid stenosis	Optovue/SQ (0–10)	Macular superficial layer	β	1.80% (1.53–2.07)	*p* < 0.001	Observational, covariate-adjusted
Czakó 2023 [6]	106 eyes/63 (MG + controls)	Optovue/SQ (0–10)	Macular superficial layer	β	0.90% (0.19–1.61)	*p* < 0.01	Observational, covariate-adjusted
Élő 2026 [17]	44 eyes/22 healthy	Optovue/SQ (0–10)	Macular VD	β	1.62% (1.23–2.02)	*p* < 0.001	Observational, covariate-adjusted
Élő 2026 [17]	44 eyes/22 healthy	Optovue/SQ (0–10)	Peripapillary all-vessel	β	0.21% (−0.27 to 0.69)	*p* = 0.39	Observational, covariate-adjusted
Élő 2026 [17]	44 eyes/22 healthy	Optovue/SQ (0–10)	Peripapillary small-vessel	β	0.13% (−0.34 to 0.60)	*p* = 0.60	Observational, covariate-adjusted
Czakó 2019 [9]	100 eyes/50 NPDR	Optovue/SSI (~0–100)	Macular superficial layer	CF	0.22% (0.20–0.24)	*p* < 0.001	Controlled experimental
Czakó 2019 [9]	100 eyes/50 NPDR	Optovue/SSI (~0–100)	Macular superficial layer	CF	0.23% (0.21–0.26)	*p* < 0.001	Controlled experimental
Lim 2018 [8]	446 eyes/healthy	Cirrus/SS (1–10)	Macular	r	r = 0.668	*p* < 0.001	Observational (correlation)
Lim 2019 [10]	259 eyes/healthy	Cirrus/SS (1–10)	Peripapillary	r	r = 0.413	*p* < 0.001	Observational (correlation)
Al-Sheikh 2017 [20]	17 eyes/10 healthy	Optovue AngioVue	Macular superficial layer	ICC	ICC 0.8 → 0.3	—	Controlled experimental
Pooled [5,6,13,17]	4 cohorts	Optovue/SQ (0–10)	Macular superficial layer	CF	1.64% (1.15–2.12)	*p* < 0.001	Mixed (controlled + covariate-adjusted), pooled

Note: Peripapillary estimates were not pooled. Czakó reported whole-image and annular correction factors from the same experimental cohort [13], whereas Élő reported observational coefficients from a separate cohort [17]; these sparse within-cohort-correlated estimates were therefore summarised descriptively. Cohort overlap: Mihály overlaps with István [4,5]; one estimate per cohort entered the primary macular pool and the other sensitivity analysis.

**Table 2:** Direct cross-sectional Pearson correlations between manufacturer-reported image quality and macular vessel-density outcomes in independent healthy cohorts. Note: Correlations were pooled after Fisher z-transformation using a DerSimonian–Laird random-effects model. Cochran Q = 3.94, *I*^2^ = 49%, and τ^2^ = 0.018. The pooled correlation is a scale-independent directional summary, not a device independent correction factor. Partial correlations, within-eye change correlations, repeated measurement associations, experimental attenuation associations, and non-VD flow outcomes were excluded from this synthesis. Non-pooled slope estimates, including the binned-mean slopes [8,10] and inter-individual slope [11] were directionally concordant with the pooled findings but were excluded from formal pooling, as they conflate physiological and inclusion-related variation with the isolated quality effect and lack reconstructable confidence intervals.

Study	Population	Device/Quality Metric	Macular Vascular Outcome	r	n
**Lim 2018** [8]	Healthy participants	Cirrus AngioPlex/signal strength (0–10)	Macular vessel density	0.668	446
**Takusagawa 2017** [26]	Healthy participants	Optovue AngioVue/signal-strength index	6 × 6 mm superficial vascular-complex vessel density, fixed-threshold analysis	0.731	30
**Xiong 2023** [27]	Healthy control participants	Optovue AngioVue/signal-strength index	6 × 6 mm commercial extrafoveal vessel density	0.434	34
**Random-effects pooled estimate**	Three independent cohorts	Non-interchangeable device-specific indices	Direct cross-sectional macular VD correlations	0.64 (95% CI 0.50–0.75)	510

## Data Availability

No new data were created or analyzed in this study. Data sharing is not applicable to this article.

## Supplementary Materials

The following supporting information can be downloaded at: https://www.mdpi.com/article/10.3390/medsci14040498/s1, Supplementary Table S1 summarises the studies included in the qualitative synthesis, together with their OCTA platform, im-age-quality metric, design, and study population.

## Abbreviations

The following abbreviations are used in this manuscript:

AMD	Age-related macular degeneration
AUC	Area under the curve
CCFD	Choriocapillaris flow deficits
CF	Correction factor
CI	Confidence interval
CV	Coefficient of variation
DCP	Deep capillary plexus
FAZ	Foveal avascular zone
GEE	Generalised estimating equation
ICC	Intraclass correlation coefficient
MG	Monoclonal gammopathy
NPDR	Non-proliferative diabetic retinopathy
OCT	Optical coherence tomography
OCTA	Optical coherence tomography angiography
OSCAR-MP	OCTA Study group Consensus for Artifacts and Reporting—Motion, Perfusion (consensus quality criteria)
PD	Perfusion density
RNFL	Retinal nerve fibre layer
SCP	Superficial capillary plexus
SD	Spectral-domain
SQ	Scan quality
SS	Signal strength
SSI	Signal-strength index
SS-OCTA	Swept-source OCT angiography
VD	Vessel density
VLD	Vessel length density
