# Supplementary material for: Image Quality as an Important Confounder in Quantitative OCT Angiography: A Review with Quantitative Synthesis
Source: Med Sci (Basel). 2026 Aug 20;14(4):498. doi: 10.3390/medsci14040498 (PMC13515158; doi:10.3390/medsci14040498)
Supplement: Supplementary file 1 [file medsci-14-00498-s001.zip › medsci-4480165-supplementary.pdf]

Supplementary Table S1 summarises the studies included in the qualitative synthesis, together with their OCTA platform, image-quality metric, design, and study population.

#	Study	OCTA platform	Quality metric (scale)	Design type	Population
1	Yu 2019	Optovue AngioVue + Zeiss Cirrus AngioPlex	SSI (0-100)	Controlled (defocus) + observational	Healthy volunteers
2	Lim 2018	Zeiss Cirrus AngioPlex	Signal Strength (0–10)	Observational (correlation)	Healthy, n = 446 eyes
3	Lim 2019	Zeiss Cirrus AngioPlex	Signal Strength (0–10)	Observational (partial correlation)	Healthy, n = 259 eyes
4	Lee 2022	Cirrus (SD-OCTA) + PlexElite (SS-OCTA)	Signal Strength (0–10)	Controlled (signal reduction, 2 devices)	Healthy, n = 20 eyes
5	Dhodapkar 2022	Not reported*	Automated 'signal strength' (comparator)	Observational / DL methodology	134 patients, 347 scans
6	Czakó 2018	Optovue AngioVue	SSI (0-100)	Observational (reproducibility)	Diabetic, n = 54 eyes
7	Czakó 2019	Optovue AngioVue	SSI (0-100)	Observational (reliability)	Diabetic (NPDR)
8	Al-Sheikh 2017	Topcon Triton (SS-OCTA)	Image Quality Index	Controlled (artificial quality degradation)	Healthy, 17 eyes/10 subjects
9	Czakó 2020	Optovue AngioVue	SQ (0–10)	Controlled experimental (determ. signal loss)	Healthy
10	Lujan 2021	Optovue AngioVue + Zeiss AngioPlex	SSI (0-100)	Observational (multicentre quality audit)	Diabetic retinopathy, 787 eyes/461 subjects
11	Lee T 2023	Not reported*	Manual good/poor quality (binary)	Observational / CNN methodology	Neurodegenerative disease
12	Lin 2024	Not reported*	Signal strength (threshold ≥ 6)	Observational (retrospective)	546 patients, 4555 images
13	Lee WH 2021	Presumed Zeiss Cirrus (SS)*	Signal Strength	Observational (long-term repeatability)	Healthy
14	Sampson 2022	N/A — review	N/A	Review / methodological overview	N/A
15	Wicklein 2023 — OSCAR-MP	Multi-platform consensus	Qualitative consensus criteria	Consensus/methodological	N/A
16	Enders 2019	Zeiss Cirrus 5000 + AngioPlex module	"Excellent"/"sufficient" (qualitative)	Observational (cross-sectional)	75 eyes, mixed retinal disease

17	Deussen 2024	Not reported*	Expert-grading (% good quality)	Observational (retrospective case series)	nAMD, 198 eyes
18	Pradhan 2022	Optovue AngioVue (assumed) *	SSI	Observational	Pseudoexfoliation syndrome
19	Jung 2020	Not reported*	No formal quality metric	Observational (cross-sectional cohort)	Retinal vein occlusion, 10 patients
20	Gujar 2025	Heidelberg Spectralis OCTA module	Qualitative expert score	Observational (protocol comparison)	Healthy, 20 eyes
21	István 2022	Optovue AngioVue	SQ (0–10)	Observational, covariate-adjusted	Carotid stenosis
22	Mihály 2023	Optovue AngioVue	SQ (0–10)	Observational, covariate-adjusted	Carotid stenosis (partial cohort overlap with István)
23	Czakó 2023	Optovue AngioVue	SQ (0–10)	Observational, covariate-adjusted	Monoclonal gammopathy
24	Magyar-Stang 2023	Optovue AngioVue (assumed) *	SQ (0–10)	Observational, covariate-adjusted	Carotid stenosis
25	Élő 2026	Optovue AngioVue	SQ (0–10)	Controlled (light-adaptation) + observational, covariate-adjusted	Healthy, functional/neurovascular coupling
26	Takusagawa 2017	Optovue RTVue XR Avanti / AngioVue	Signal-strength index (SSI, approximately 0–100)	Observational cross-sectional correlation; fixed-threshold and reflectance-compensated analyses	Healthy participants, n = 30 eyes
27	Xiong 2023	Optovue RTVue XR Avanti / AngioVue	Signal-strength index (SSI, 0–100) and shadow area	Observational cross-sectional correlation; algorithm-comparison study	34 healthy controls and 118 participants with diabetes, one eye per participant
